# Evolutionary development of the amygdaloid complex

**DOI:** 10.3389/fnana.2013.00027

**Published:** 2013-08-28

**Authors:** Mohan Pabba

**Affiliations:** Neurosciences Unit, Department of Cellular and Molecular Medicine, Faculty of Medicine, University of OttawaOttawa, ON, Canada

**Keywords:** amygdala, anatomy, tetrapods, mammals, evolution

In the early 19th century, Burdach discovered an almond-shaped mass of gray matter in the anterior portion of the mammalian temporal lobe, which he called “amygdala” (Burdach, 1819–1822). The first anatomical description of the amygdala was made in 1867 by Meynert (1867). Subsequently, a large number of other nuclei were added to the amygdala to constitute what is now known as the “amygdaloid complex” (AC) (Johnston, 1923). Until this day, AC remains a subject of intense investigation in terms of content and evolutionary development since it is a much more complicated structure than what was previously thought. It is therefore, important to know the evolutionary developmental origin of AC before we can completely understand its function.

The AC is a multinuclear complex comprised of 13 nuclei. These nuclei are divided into three major groups: the basolateral, cortical-like, and centromedial. Other accessory nuclei such as the intercalated cell masses (I) and the amygdalo-hippocampus area have also been described. The basolateral group is comprised of the lateral nucleus (LA), basal nucleus (B), and accessory basal nucleus (AB) (Johnston, 1923). The cortical-like group of nuclei includes the nucleus of the lateral olfactory tract (NLOT), bed nucleus of the accessory olfactory tract (BAOT), anterior and posterior cortical nuclei (CoA and CoP, respectively), and periamygdaloid cortex (PAC). The centromedial nucleus consists of the central nucleus (CeA), medial nucleus (M), and amygdaloid part of the bed nucleus of stria terminalis (BST). The major remaining groups of AC are the amygdalohippocampal area (AHA) and intercalated nuclei (I) (Aggleton, 2000; Sah et al., 2003). These different nuclei of AC are connected within and also with various brain regions, and thus, process various types of information (e.g., olfactory and Figure 1A).

**Figure 1:**
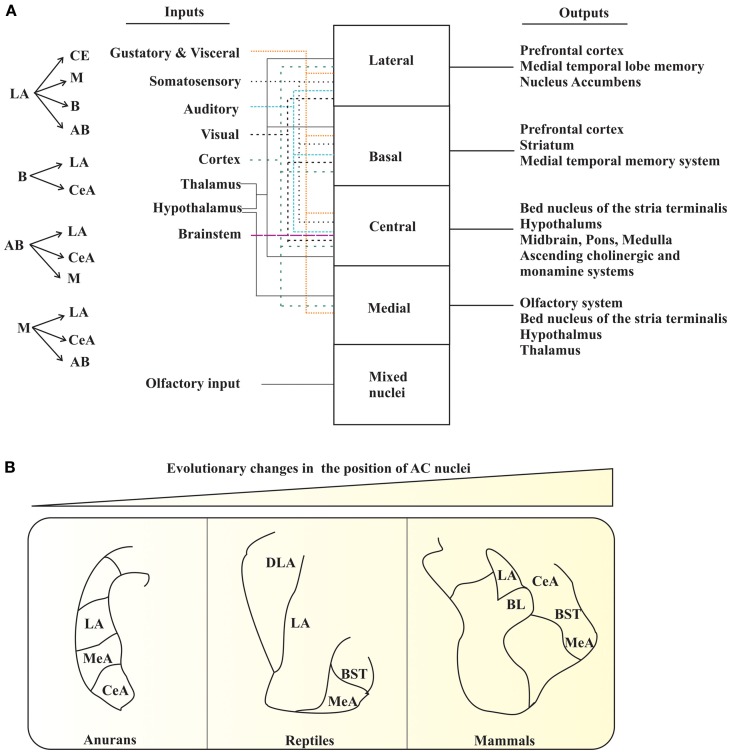
**AC of tetrapods. (A)** The interconnections (*left panel*) and summary of the main inputs to AC and outputs from AC (*right panel*). The various types of inputs to AC are denoted with different types of colored arrows. **(B)** Pictorial diagram depicting the changes in the position of certain AC nuclei during the course of evolution. LA, lateral nucleus; MeA, medial nucleus; CeA, central nucleus; DLA, dorsal division of lateral nucleus; BST, bed nucleus of stria terminalis; BL, basolateral amygdala. The figure panels **(A)** and **(B)** are adapted and modified from Sah et al. (2003); Moreno and Gonzalez (2007b).

Swanson and Petrovich (1998) definition of amygdala as neither a structural nor a functional unit provides an attractive point to explore the evolutionary developmental aspects of AC because a growing number of evidence suggests AC as an evolutionarily conserved structure. Earlier, research on structural organization of AC in different amniotic vertebrates revealed a common pattern of organization, along with shared functional roles. Conversely, research on anamniotes provided little comparative information regarding structural organization of AC. However, recent studies have shown a homology between amygdaloid components of amniotes and anamniotes. To better understand the evolutionary and developmental history of a particular brain region, one needs to follow a “sequential (step by step) approach,” which takes into account the developmental, topological, hodological, genetical, and functional history. Interestingly, recent data on AC of mammals, reptiles, and anurans suggest that the evolution of AC occurred as common traits of telencephlon, for example, regions of cortical amygdala such as nLOT and accessory olfactory bulb (AOB) (Remedios et al., 2007; Huilgol et al., 2013); but not as the sum of unrelated structures with different origins. The present understanding of AC in developmental and adult vertebrates suggests two major divisions of telencephlon: the pallium and the subpallium (Puelles et al., 2000; Martinez-Garcia et al., 2002; Moreno and Gonzalez, 2007b; Remedios et al., 2007). This dual view or origin makes AC a histogenetic complex structure of the adult brain, with extremely intense morphogenetic and migratory processes during the development in all tetrapods (Puelles et al., 2000). In mammals, the pallial component is composed of “cortical amygdala” and “basolateral amygdala.” In turn, the subpallial component consists of the striatal component, central amygdala, and medial amygdala. This basic plan is shared by reptiles, birds, and also by anuran amphibians (Martinez-Garcia et al., 2002; Medina et al., 2004). Interestingly, this basic description is possible only in few mammalian tetrapods, but not in the non-mammalian amniotes and the anurans where they have no clear anatomical subdivisions. The existence of shared embryological AC components in all tetrapods provides clues to the presence of precursors of the amygdaloid nuclei from anamniotes (Moreno and Gonzalez, 2007b). The following sections deal with the current view on accepted and shared components of AC in tetrapods.

The amygdala is a part of a phylogentically conserved olfactory system, particularly the olfactory bulb, in vertebrate evolution in terms of embryological origin, neurochemistry, connectivity, and function (Martinez-Garcia et al., 2002; Huilgol et al., 2013). Additionally, a major part of amygdala is also an integral component of the vomeronasal system of the tetrapod (except avian) brain (Swanson and Petrovich, 1998; Moreno and Gonzalez, 2005a).

In mammals, the vomeronasal information passes via the AOB to medial (MeA) and cortical postero-medial amygdala (CoApm) (Swanson and Petrovich, 1998). In addition, the amygdala also receives information from the main olfactory bulb (MOB) and hypothalamus to modulate reproductive and defensive behaviors (Canteras et al., 1995). In reptiles and anurans, the existence of a well-developed “vomeronasal amygdala” has also been reported (Moreno and Gonzalez, 2003), although no vomeronasal amygdaloid nuclei has been described in birds so far (Martinez-Garcia et al., 2006). Thus, in all tetrapods, the main secondary vomeronasal brain areas belong to AC.

In mammals, the olfactory amygdaloid system consists of the distinct cortical (CoA, CoP nuclei, etc.) and basolateral amygdala (BL, M, LA nuclei). LA receives major sensory input, and is important for emotional behavior (Ledoux et al., 1990). Studies on birds indicate the presence of nuclei that are comparable to CoP of the mammalian amygdala. These studies also revealed the possession of counterparts similar to BM and LA of the mammalian amygdala (Martinez-Garcia et al., 2006). In reptiles, studies on the olfactory system showed comparable functional circuitry with the mammalian BA complex. The anuran counterpart of the mammalian olfactory amygdala is LA (Moreno and Gonzalez, 2004). This part of the amygdala in anurans receives directly or indirectly olfactory, visual, auditory, somatosensory, vomeronasal, and gustatory information. The observed integration that occurs in AC of tetrapods is responsible for the acquisition of “emotional memory,” which pertains to the survival of individuals during their defence against danger, their interaction with sexual partners, or their fight with an enemy (Ledoux, 2000). Therefore, the amygdala receives large sensory input information from olfactory and vomeronasal projections, and are conserved in tetrapods.

Studies on tetrapods showed CeA as the main receiver of a wide range of sensory information from other amygdaloid regions in addition to the thalamus and brain stem. Moreover, CeA is known to link and integrate the emotional and motor components of behavior (Han et al., 1997), behavioral responses to nociceptive and visceral pain (Han and Neugebauer, 2004), and behavioral responses to stressful stimuli (Saha et al., 2000). CeA also mediates many of the autonomic, somatic, endocrine, and behavioral responses in different tetrapods. The autonomic amygdala provides a link between environmental stimuli and animal behavioral responses, and thus, provides an important significance in terms of evolutionary conservation.

Another conserved shared system of tetrapods is the strong amygdalo-hypothalamic connections (Martinez-Marcos et al., 1999; Moreno and Gonzalez, 2005a). In mammals, nuclei that project to hypothalamus through the stria terminalis arise from the medial and basolateral amygdala (Swanson and Petrovich, 1998). As in mammals, the amygdalo-hypothalamic projections of anurans (Moreno and Gonzalez, 2005b) also project through the stria terminalis. In anurans, the amygdalo-hypothalamic connections control functions mediated by the hypothalamus in response to pheromones and odors (Reiner and Karten, 1985; Swanson and Petrovich, 1998).

As in mammals, the amygdalo-hypothalamic projections of anurans, project through the stria terminalis. The main similarity with amniotes is the projection to the hypothalamus from comparable amygdaloid territories carrying vomeronasal, olfactory, and multimodal information (Reiner and Karten, 1985; Swanson and Petrovich, 1998). The situation of amygdalo-hypothalamic projections in birds is more complicated because of the lack of a well-developed olfactory/vomeronasal system.

On the other hand, studies that compare the distribution of neuronal markers (either proteins or genes/transcription factors) across the development of analogous AC nuclei from different species as well as within the same species have also provided valuable information on the evolution of AC. For instance, similarities in the molecular profiles of the pallium and subpallium of mice and chickens were obtained by comparing the nested expression domains of genes such as Dlx-2, Tbr-1, Pax-6, NKx-2.1, and Emx-1 (Puelles et al., 2000). Moreno and Gonzalez, using the distribution of somatostatin, nitric oxide synthase, etc. in anuran CeA and MeA in a series of studies, has predicted that these parts of AC could be related to AC of amniotes (Moreno and Gonzalez, 2005a, 2007a). Medina et al. demonstrated a possible existence of evolutionary relationship in various AC nuclei of mammals, reptiles and birds by testing the expression patterns of genes/transcription factors such as Lhx2, Lhx9, Pax6, Islet 1, NKx2, Lhx6, and Lhx5 in forebrain regions of these animals (Medina et al., 2011). Using the similarities and differences in the expression of Lhx1 and Lhx5, Abellan et al. suggested a common pattern of evolutionary conservation in telencephalon between mice and chickens during various stages of development (Abellan et al., 2010). By examining the distribution of Lhx2, Trb1, reelin, and CdK5, Remedios et al. estimated a possible developmental and evolutionary link between nLOT of AC and neocortex (Remedios et al., 2007; Subramanian et al., 2009). Another study from Tole's group, using migratory genetic markers (e.g., NP2 and AP2α), showed that the distinct halves of AOB [posterior and anterior (pAOB and aAOB)] has different developmental origins, and that pAOB could be a component of AC as it was positioned closely to MeA in anamniotes (*Xenopus*) (Huilgol et al., 2013). Finally, Barger et al. using a different approach, i.e., by comparing the percentage of neurons in individual nuclei of AC between humans and apes, suggested that during the course of human evolution, LA of AC has further progressed in humans (Barger et al., 2012).

## Concluding remarks

The classical hypothesis proposed by Edinger regarding the evolution of the brain attracted much attention (Edinger, 1908). He proposed that the telencephlon evolved in progressive stages of complexity and size, culminating to the human brain. He also stated that there is an “old brain” (the subpallium at the telencephalic base) followed by the addition of a “new brain” (the pallium at the top of the telencephlon). Nevertheless, this classical hypothesis provides evidence on the existence of a basic plan in the origin, regionalization, and organization of the forebrain of vertebrates. Based on the data pertaining to the organization of AC, there seems to have several important features that are common to all tetrapods: (1) it is formed by pallial and subpallial derivatives; (2) it is topographically situated in the ventrolateral caudal telencephalic hemispheres; (3) it has shared features in relation with different functional systems like the vomeronasal, olfactory, autonomic, and multimodal systems along with an intricate intra-amygdaloid network; (4) it is the origin of important hypothalamic projections; (5) it has a common embryological origin for several prominent features of AC; (6) it has the presence of a main output for autonomic system; finally, (7) it has abundant local circuit neurons that are shared by most amniotes. Thus, in the light of recent findings on AC (Remedios et al., 2007; Butler et al., 2011; Huilgol et al., 2013) also strongly support the idea that tetrapods share the same basic plan.

The increase in size of the pallium, especially in mammals, has an evolutionary importance. The current spatial arrangements of the mammalian AC are still found in living anurans. Therefore, it is now obvious that these “new evolutionary nuclei” would have pushed the “most conserved nuclei” (Moreno and Gonzalez, 2007b). It explains why mammals have the central, medial, and basolateral nuclei occupying the most medial positions, whereas the cortical amygdaloid nuclei occupy the most lateral positions (Figure 1B) (Moreno and Gonzalez, 2007b). Consequently, the brain of ancestral tetrapods developed an elaborate AC in response to new requirements imposed on them as a part of the transition from water-to-land. Therefore, the basic organization of the brain system, at least in the case of the AC, is still recognizable in all existing tetrapods, and can be compared with that of mammals.
